# Arthroscopic treatment of focal osteochondral lesions of the first metatarsophalangeal joint

**DOI:** 10.1186/s13018-017-0562-7

**Published:** 2017-04-27

**Authors:** Ersin Kuyucu, Harun Mutlu, Serhat Mutlu, Baris Gülenç, Mehmet Erdil

**Affiliations:** 1 Orthopedics and Traumatology, Istanbul Medipol University, Istanbul, Turkey; 2 Orthopedics and Traumatology, Taksim Ilkyardım Training and Education Hospital, Istanbul, Turkey; 3 Orthopedics and Traumatology, Kanuni Sultan Süleyman Training and Education Hospital, TEM Avrupa Otoyolu Göztepe Çıkışı No:1, Bağcilar, Istanbul, Turkey

**Keywords:** Metatarsophalangeal joint, Hallux rigidus, Arthroscopy

## Abstract

**Background:**

Although arthroscopic surgical treatment of the first metatarsophalangeal (MTP) joint involves painful sesamoid excision, synovectomy, debridement, and partial cheilectomy, no gold standard treatment technique has been defined in the literature for hallux rigidus and focal osteochondral lesions. This study aimed to assess the arthroscopic treatment for early grade focal osteochondral lesions of the first MTP joint and to determine the impact of arthroscopic microhole drill surgery on foot function and activities of daily living in a group of patients who failed conservative treatment.

**Methods:**

This prospective study included 14 patients with hallux rigidus and focal osteochondral lesions of the first MTP joint who underwent surgery in 2014 and were followed on a regular basis thereafter.

**Results:**

The patients had mean preoperative VPS (visual pain score) and AOFAS (American Orthopedic Foot and ankle Society)-Hallux scores of 8.14 ± 0.86 SD and 48.64 ± 4.27, respectively; the corresponding postoperative values of both scores were 1.86 ± 0.66 SD and 87.00 ± 3.70. Both VPS and AOFAS-Hallux scores changed significantly.

**Discussion:**

In this prospective study, we explored the impact of arthroscopic microhole drill surgery on foot function and activities of daily living in patients with focal osteochondral lesions of the first MTP joint. Our results showed significant improvements in VPS and AOFAS scores with this treatment.

**Conclusions:**

An arthroscopic microhole drill technique can be used with impressive functional scores and without any complications in patients who failed conservative therapy for hallux rigidus with focal chondral injury.

## Background

First defined by Watanabe in 1972 [1, 2], arthroscopic treatment of the first metatarsophalangeal (MTP) joint was later detailed by Barlett in 1988 [3]. Thanks to advancements in arthroscopic techniques and technology, arthroscopy of the first MTP joint is now used for both diagnosis and treatment of a variety of clinical conditions, such as hallux valgus, gout, and hallux rigidus [4]. Although arthroscopic surgical treatment of the first MTP joint involves painful sesamoid excision, synovectomy, debridement, and partial cheilectomy, no gold standard treatment technique has been defined in the literature for hallux rigidus and focal osteochondral lesions.

This study aimed to assess arthroscopic treatment, one of the surgical treatment options for early grade focal osteochondral lesions of the first MTP joint, and determine the impact of arthroscopic microhole drill surgery on foot function and activities of daily living in a group of patients who failed conservative treatment.

## Methods

This prospective study included 14 patients with hallux rigidus and focal osteochondral lesions of the first MTP joint who underwent surgery in 2014 and were followed on a regular basis thereafter. An initial recommendation for conservative treatment composed of footwear modification, analgesic use, and physical therapy for at least 6 months was offered to all patients. Patients who failed this conservative therapy, had pain, showed full-thickness cartilage injury on magnetic resonance imaging (MRI), and were followed on a regular basis participated in the study after providing written informed consent. Patients with Coughlin-Shurnas Grade-4 hallux rigidus, osteochondral kissing lesions, or an indication for osteotomy and/or cheilectomy apart from arthroscopy were excluded, as were those who did not attend regular follow-up visits. Patients were also excluded if they underwent any foot operation or had another foot deformity such as flat foot, excessive foot pronation, or moderate or severe hallux valgus. All patients were operated on by surgeons (M.E, E.K) experienced in their field. The mean postoperative follow-up period was 16.43 ± 1.86 SD months. Eight (57.1%) patients were female and 6 (42.9%) were male. The median age of the patients was 44.07 years (range, 38–49).

Coughlin and Shurnas Classification [5] was used to determine the hallux rigidus grade; Outerbridge Classification [6] was used to grade cartilage lesions. Foot function before and after surgery was assessed by the American Orthopedic Foot and Ankle Society Score (AOFAS) [7]. Visual pain scale (VPS) was used for rating pain [8]. All patients were informed about the study and provided written informed consent.

### Statistical analysis

Statistical analyses were done with the NCSS 2007 (Number Cruncher Statistical System, Kaysville, Utah, USA) software package. Descriptive statistics included mean, standard deviation, median, frequency, ratio, minimum, and maximum. Intra-group comparison of non-normal distribution parameters was done with the Friedman Test, and post hoc paired comparisons were done with the Wilcoxon signed-rank test. A *p* value of less than 0.01 was considered statistically significant.

### Surgical technique

**Fig. 1:**
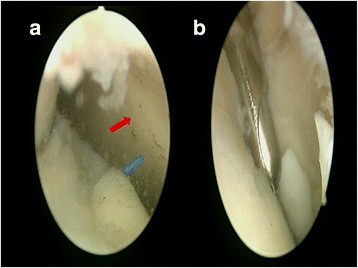
**a** First view of the MTP joint by the 30° scope. *Blue arrow* is the metatarsal head. *Red arrow* is the proximal phalangeal joint surface. **b** Synovectomy and debridement of the joint by the shaver

**Fig. 2:**
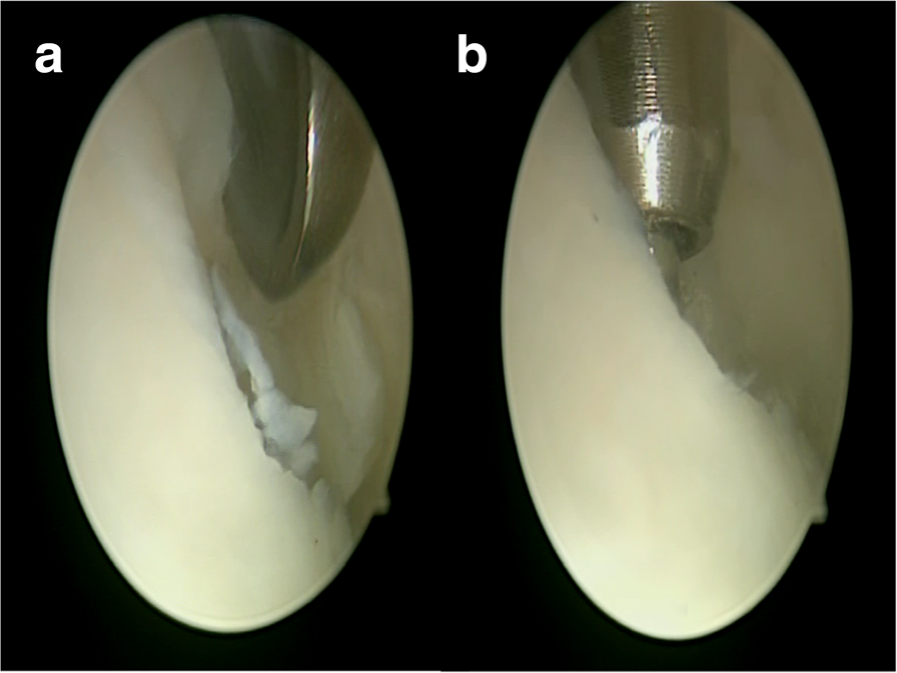
**a** Curettage of the chondral lesion. **b** Microhole drill of the chondral lesion

**Fig. 3:**
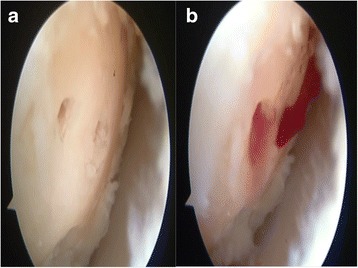
**a** Chondral surface after the microhole drill. **b** Migration of the blood from the tunnels

**Fig. 4:**
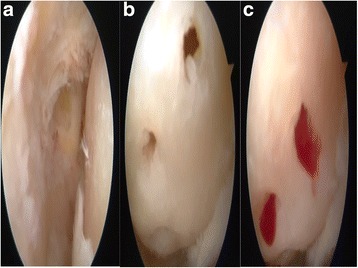
**a** Chondral defect. **b** Chondral surface after the microhole drill. **c** Migration of the blood from the tunnels

Patients were administered spinal anesthesia and placed in a supine position. An arthroscopic intra-articular water pressure system with pump was considered sufficient for hemostasis, and thus, no pneumatic tourniquet was used. A non-invasive joint distraction technique was applied. Before marking arthroscope entry points, 2–3 cc isotonic saline was administered intra-articularly to ensure capsule retention. Standard dorsolateral and dorsomedial portals were established 2–4 mm medial and lateral to the extensor hallucis longus tendon, and no additional portals were required. First, the dorsomedial portal site at the level of the joint was determined with the help of a needle. Following skin incision using a No. 15 scalpel, the joint was accessed by blunt soft tissue dissection using a hemostat, and it was visualized with a 2.0-mm 30° oblique arthroscope (Fig. 1). The dorsolateral portal was prepared with the same technique, and the arthroscope and manual devices were alternatively used through both portals. A 2.0 shaver, probe, and a straight punch were used as the manual devices. A synovectomy was done first to have a better view of the surgical field (Figs. 1 and 2). The cartilage was examined with the probe, and the joint was irrigated with abundant isotonic saline. After measuring the lesion size, the cartilage was intervened using the microhole drill method (Figs. 3 and 4). After ensuring adequate bleeding, the procedure was terminated, and the entry portals were sutured with 3/0 rapid suture.

Postoperative joint loading was permitted to the maximum tolerated point. Active and passive joint movements were not restricted on the first postoperative day. All patients were discharged 1 day after surgery. Control examinations were performed at 3 and 6 weeks and at 3, 6, 12, and 18 months.

## Results

This study included 27 patients with hallux rigidus and osteochondral injury of the first MTP joint who underwent arthroscopic surgical treatment of the first MTP joint. Five patients had Coughlin-Shurnas Grade-4 hallux rigidus and were excluded from the study, five patients were excluded due to having an osteochondral kissing lesion, and three patients were excluded for not having attended regular follow-up after the third month. After excluding the above patients, the study was completed with 14 patients. Six patients were male, and eight were female. The mean age was 44.07 ± 3.40 years. The mean follow-up duration was 16.43 ± 1.86 months. All patients were operated on by two experienced surgeons (M.E., E.K.).

The mean hallux valgus angle was 13.29° ± 1.93 SD, and the mean intermetatarsal angle was 9.14° ± 0.86 SD. Apart from joint arthroscopy, no soft tissue procedure or any procedure requiring osteotomy was done on any patient. The median operative duration was 27.8 min (range, 19–56). While nine patients had Coughlin-Shurnas Grade-2 hallux rigidus with moderate pain and joint flattening affecting less than 50% of the joint, five patients had Grade-3 hallux rigidus with severe pain and joint stiffness. Arthroscopically, all patients had Outerbridge Grade 4 full-thickness cartilage injury. Only one patient had diabetes mellitus controlled with oral antidiabetic medication.

The patients had mean preoperative VPS and AOFAS-Hallux scores of 8.14 ± 0.86 SD and 48.64 ± 4.27, respectively; the corresponding postoperative values of both scores were 1.86 ± 0.66 SD and 87.00 ± 3.70. Both VPS and AOFAS-Hallux scores changed statistically significantly (*p* < 0.01).

None of the patients developed postoperative complications.

## Discussion

**Fig. 5:**
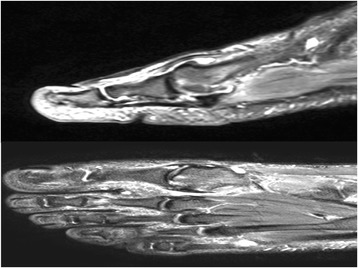
MRI of the 39-year-old man right foot, sagittal and axial view of the MTP joint

In this prospective study, we explored the impact of arthroscopic microhole drill surgery on foot functions and activities of daily living in patients with focal osteochondral lesions of the first MTP joint (Fig. 5). Our results indicated significant improvements in VPS and AOFAS scores with this treatment.

Hallux rigidus is the most common first-line pathology of the foot after hallux valgus [9]. This condition affects people at a younger age than hallux valgus, and the primary complaints are pain and movement limitations [9, 10]. Pain is usually the first symptom in the initial stages of hallux rigidus. At this stage, conservative therapy consisting of nonsteroid anti-inflammatory drug (NSAID) use and footwear modification aims to suppress synovitis and joint inflammation and reduce pain [10–12]. However, surgical treatment may be preferred when conservative therapy fails in these young and active patients [11–14]. We first applied conservative treatment in the whole patient group, and we proceeded with surgery when that treatment failed. Hallux valgus can be managed via open or arthroscopic surgery, depending on patient characteristics and disease stage [14, 15] Derner and Aldo showed that arthroscopic surgery was more beneficial than open surgery by being minimally invasive, having a shorter recovery period, requiring no special rehabilitation program, and allowing patients to return to daily activities quickly [16–18]. We achieved fast return to work with the arthroscopic approach in suitable patients since this approach caused no complications and allowed joint loading with as much weight as tolerated on the same day of surgery as mentioned in the literature [16, 17]. Pain reduction is another important marker for treatment. Our study demonstrated significantly lowered VPS scores at both first month follow-up and the last control visit compared to the preoperative period in a homogenous patient population involving only patients with hallux rigidus.

Arthroscopic treatment of the MTP joint is used alone or in conjunction with metatarsal osteotomies for the treatment of a wide array of conditions such as synovitis, hallux rigidus, gout, and degenerative hallux valgus [16]. However, there is still insufficient information about the principles of the evaluation and treatment of these patients. Whereas, three patients in Anh’s study [17] and five patients in the series published by Van Dijk [18] had this condition, no study to date has specifically addressed it. Moreover, there is no study that addresses patients having hallux rigidus with only focal osteochondral lesions.

Satisfactory improvement of hallux rigidus has been reported with arthroscopic cheilectomy and debridement without a need for revision [19]. However, the goal of this technique is to remove and clear only the injured part that restricts motion, but not to treat other intra-articular pathologies. As the potential for spontaneous healing is slim in the case of chondral injury, treatment with either the microfracture or microhole drill techniques should be considered. The microhole drill technique we used in this study is based on the principle of opening 4–6-mm-long tunnels to enable stem cells to migrate to the injured area and achieve healing with differentiation in full-thickness chondral injuries with exposed subchondral bone [20, 21]. As it was previously reported that the thickness and quality of the newly formed cartilage are better with the microhole drill technique than with the microfracture technique [21], we chose the former technique. The most notable indication of the success of this surgical technique was the ability of the patient to use his/her foot with comfort in daily activities; likewise, we demonstrated significant increases in AOFAS scores following surgery.

Postoperative complications are another problem. While a secondary surgery may be required after open surgeries, especially when cheilectomy is selected [22], complications necessitating additional surgery may occur at a rate of 6% after arthroplasty or arthrodesis [23]. Lin and Murphy’s trial reporting clinical-radiological progression and numbness in the first web space at a rate as high as 40% after cheilectomy in a patient group with hallux rigidus indicates the fact that surgical procedures that appear simple may not be as innocuous as perceived [24]. No minor or major complications occurred at early or late periods with our arthroscopic microhole drill technique; rather, there occurred a rapid relief of pain and a quick return to daily activities.

The strongest aspects of our study are its prospective cohort study design and a homogenous study sample. Our most notable limitations, on the other hand, are the lack of a comparison group with hallux rigidus managed by either open surgery or conservative therapy, as well as a short follow-up period. Another limitation of our study is its small sample size.

## Conclusions

In conclusion, an arthroscopic microhole drill technique can be applied with impressive functional scores and without any complications in persons who failed conservative therapy for hallux rigidus with focal chondral injury. There is a need for comparative studies with long-term follow-up in this patient population.

## Abbreviations

AOFAS: American Orthopedic Foot and Ankle Society Score
MTP: Metatarsophalangeal
NSAID: Nonsteroidal anti-inflammatory drug
VPS: Visual pain scale
